# Ten simple rules for developing a reporting appraisal tool

**DOI:** 10.1371/journal.pcbi.1014841

**Published:** 2026-10-16

**Authors:** Kyle Morrison, Malgorzata Lagisz

**Affiliations:** Collaboration for Open Science and Synthesis in Ecology and Evolution (COSSEE), Department of Biological Sciences, University of Alberta, Edmonton, Alberta, Canada; Dassault Systemes BIOVIA Ltd, UNITED STATES OF AMERICA

## Abstract

In an ideal world, authors would report clearly and transparently exactly what they conducted. In reality, reporting is often incomplete, and key elements of research conduct are frequently missing from manuscripts. Reporting guidelines were developed to address this problem by helping authors identify what should be reported. These guidelines have been highly valuable for authors, but they are not well suited for appraising reporting during peer review or editorial assessment despite common use. This is because authors and appraisers have different needs: authors require a comprehensive guide for writing, whereas appraisers need a tool that is concise, repeatable, and easy to use. We therefore propose the development of reporting appraisal tools: operationalised tools designed specifically to assess whether a paper has reported the minimum essential elements of a study. To support their development, we present 10 simple rules.

## Introduction

In an open research utopia, authors would report exactly what they conducted in a clear and transparent manner. However, this ideal is rarely achieved [1], creating challenges for readers, editors, and reviewers who must judge a study’s reliability and robustness [2]. These challenges arise because poor reporting can conceal important flaws and biases [1]. Put simply, readers cannot assess what they cannot see, and they cannot see what authors do not report. To improve reporting practices, numerous reporting guidelines have been developed [3]. These guidelines provide practical sets of items that should be reported in a manuscript describing a given type of research [3]. Well-known examples include PRISMA for systematic reviews [4] and CONSORT for randomised controlled trials [5]. Today, such guidelines are among the most cited publications of all time and are endorsed by many highly reputable journals [6].

Using reporting guidelines is highly valuable for authors writing their manuscripts [3], but they should not be used to judge the completeness of reporting in manuscripts written by others, such as during peer review. This is primarily because authors and appraisers have different needs: authors need a comprehensive checklist to guide reporting, whereas appraisers require a tool that is representative, consistent, and easy to use [7]. In other words, authors benefit from a detailed list covering all reporting items, while appraisers need a more concise tool that captures the essential items in a format they can apply repeatedly and can compare outcomes across studies.

Consequently, appraisers interested in reporting completeness require dedicated tools that are operationalised and fit for this specific use case. We term these r*eporting appraisal tools*. They can be used to indicate which predefined key elements of a study were described in a manuscript, at least at a minimum level, and which were not, revealing important gaps in reporting [7]. Despite the growing demand for tools to appraise reporting completeness and research transparency, specifically designed tools remain rare. There is therefore a clear need to promote their development.

Historically, developers of reporting guidelines have had to generate reporting items de novo, a process that is often complex, resource-intensive, and time-consuming. By contrast, *reporting appraisal tools* can be developed more efficiently by drawing on existing reporting guidelines, which already provide curated and conceptually refined sets of reporting items. Adapting these items into appraisal questions can reduce duplication of effort and conserve resources, while building on work that has already undergone substantial development.

Beyond the initial adaptation of items, developers of *reporting appraisal tools* should follow the robust, consensus-based methods recommended for reporting guideline development [3,8], while also considering usability, interpretability, and repeatability, as is common in the development of traditional certainty assessment tools, critical appraisal tools, and risk of bias tools such as GRADE-related tools [9], AMSTAR2 [10], and ROBIS [11]. Importantly, *reporting appraisal tools* are not intended to be an additional bureaucratic burden layered onto existing checklists. Instead, they are shorter, operationalised derivatives of existing reporting guidelines, tailored to appraisal rather than manuscript writing.

The rules presented below are largely informed by our own experience developing MATES, a reporting appraisal tool for meta-analyses [7], and by our methodological analysis of PRISMA extension development processes [6]. We therefore present this article as a synthesis grounded in experience with reference to the reporting guideline and critical appraisal development literature. To support the development of reporting appraisal tools, we present 10 simple rules (Fig 1).

**Fig 1:**
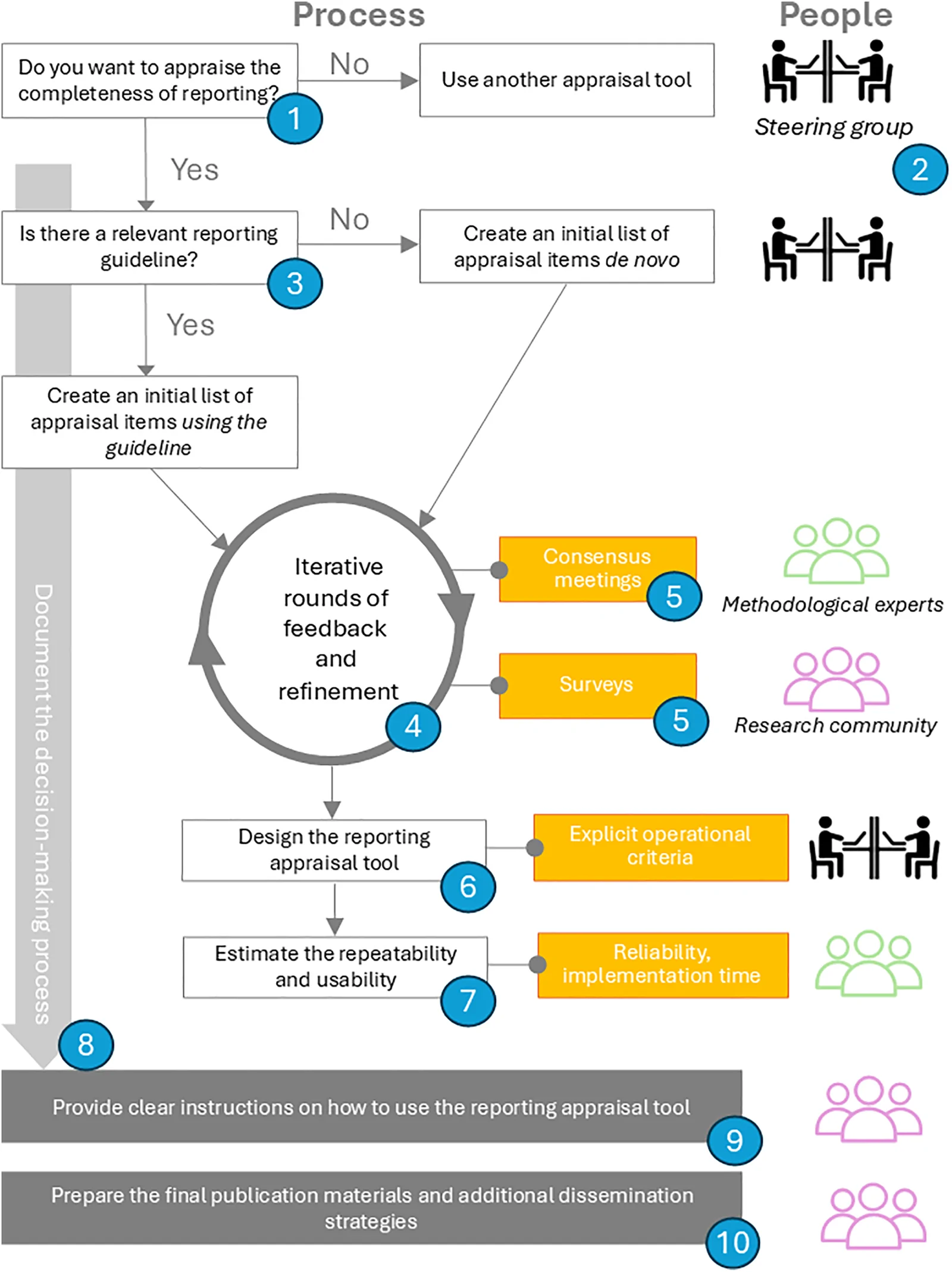
An overview of the 10 simple rules. The numbers depicted in the blue circles refer to the relevant rule numbers in the text.

## Rule 1: Reporting completeness is distinct from methodological quality

A fundamental first step when creating any tool is to define exactly what you aim to assess [8]. In this article, we present rules to develop tools to appraise the completeness of reporting, drawing from established reporting guidelines. This is distinct from appraisal tools developed to appraise methodological quality or risk of bias (e.g., AMSTAR2 [10]). Appraisal tools designed to assess reporting completeness can be used by editors, reviewers, and readers to evaluate whether a manuscript sufficiently describes what was done, without judging the study’s design [7]. The following set of rules share conceptual overlap with established development methodologies conducted to develop reporting guidance such as conducting a structured, iterative, consensus-based development, and those of more traditional critical appraisal tools of methodological quality such as estimating reliability and usability metrics.

## Rule 2: Assemble a steering group to guide development

The next step in developing a reporting appraisal tool is to assemble a core team to lead the process [8]. This team, often referred to as the steering group, should include individuals with the methodological expertise, topic knowledge, and practical insight needed to guide the tool’s development. In forming the group, it is important to consider not only technical expertise, but also whether the team reflects a range of perspectives relevant to the tool’s intended use. The steering group is responsible for defining the scope of the appraisal tool, designing the development process, and overseeing how feedback will be sought and incorporated [3]. It should coordinate the overall process, organise and synthesise feedback, document key decisions, and make subsequent revisions to the tool. Once the tool is finalised, the steering group should also lead preparation of the manuscript and associated materials, ensuring that the development process is reported transparently and in sufficient detail to support trust, uptake, and future updating (see Rules 8, 9, and 10 for more details).

## Rule 3: Select a suitable reporting guideline as a foundation

Once you decide to develop a tool to appraise the completeness of reporting, and assemble your steering group, the next step is to identify a suitable reporting guideline to serve as a source of candidate assessment items. You can do this by first specifying the field, study type, and study design for which you want to appraise reporting completeness [9]. Once these elements are specified, you can begin searching for a reporting guideline which aligns most closely. A useful starting point is to look at established databases such as the one collated by the EQUATOR network—it neatly classifies almost 700 existing guidelines based on their scope and methodology [3]. You can also search across items stored in banks of evaluation items, such as those described in [12]. If no reporting guideline closely matches your needs, you may need to select a guideline that can be adapted or extended to add specific items required for your specific needs.

## Rule 4: Incorporate diverse expert and community opinion

An important consideration is whose perspectives are needed to inform the process effectively. Typically, this involves engaging a combination of methodological experts, relevant stakeholders, and members of the broader community. As with assembling the core team, it is important to look beyond disciplinary expertise alone—consider relevant PROGRESS-Plus factors that may shape participants’ perspectives [13]. Including participants from low- and middle-income countries can surface critical perspectives on the feasibility of mandating resource-intensive reporting practices [14]. This is especially important when developing reporting appraisal tools, as it is counterproductive to appraise the reporting of elements that are rarely conducted in practice. Bringing together diverse expertise ensures feedback that is well-rounded, representative, and informative for tool development.

## Rule 5: Gather feedback iteratively using complementary methods

The item selection process should use a structured, transparent procedure to identify the most important reporting items in a guideline and to clearly differentiate these from more peripheral, nonessential items. An effective approach for the item-selection procedure is a Delphi-like approach which uses iterative rounds of feedback from the research community and from methodological experts [15]. These iterative rounds can combine complementary methods such as consensus meetings and surveys [15]. These complementary approaches allow for collection of both qualitative and quantitative feedback which can help determine the best course of action. Another strength of a Delphi-like approach is that it enables the involvement of a diverse group of participants including a broad range of nationalities, genders, career stages, as well as other PROGRESS-Plus factors [13]. Therefore, a Delphi-like approach can be used to refine reporting items in a checklist whilst garnering the perspectives of a broad audience representative of the communities’ opinions. As broader stakeholder involvement can increase the number of candidate items as participants advocate for domain-specific additions, the steering group should pre-specify item-retention thresholds before the Delphi process begins (e.g., retaining only items reaching a defined consensus threshold, such as 70% agreement) and consider a dedicated prioritisation round—such as forced ranking or a pre-agreed cap on total item count—to filter the candidate pool. A subsequent piloting stage (see Rule 7) can further identify items that are redundant or contribute negligible discriminatory value, allowing the steering group to remove them prior to finalisation. Once the development methodology has been designed, each member of the steering group should formally confirm that it effectively addresses and fulfils all stated aims.

## Rule 6: Design the structure of the reporting appraisal tool

As noted above, reporting appraisal tools serve a distinct purpose from reporting guidelines and therefore require different design considerations. These differences primarily reflect the contrasting demands of appraising a study authored by others versus following a guideline for one’s own work. The first key distinction is that a reporting appraisal tool should present items as direct, answerable questions rather than as guideline-style statements instructing authors on what to report. The second is that each question should be accompanied by explicit operational criteria specifying what constitutes a “fulfilled” item (Fig 2). Providing clear, answerable questions with explicit criteria reduces ambiguity about what is required to fulfil each item and in turn enhances the consistency of appraisal outcomes [7]. For example, the guideline item “report eligibility criteria for study inclusion” could be converted into two appraisal questions with different scoring structures. As a scaled item, “Does the manuscript state inclusion and exclusion criteria?” can be rated yes (both criteria types reported with sufficient detail to replicate), partial (only one type reported), or no (neither reported). As a binary item, a related but narrower question, “Does the manuscript state the eligibility criteria”, only allows a yes/no answer, since this element is either stated or absent, with no meaningful partial state. Scaled items are best suited to be graded requirements, whereas binary items suit single, discrete reporting elements. Regarding scoring more broadly, a simple binary (yes/no) system can promote repeatability and ease of use, while scaled-response options (e.g., yes/partially/no) are better suited when meaningful gradations exist [10].

**Fig 2:**
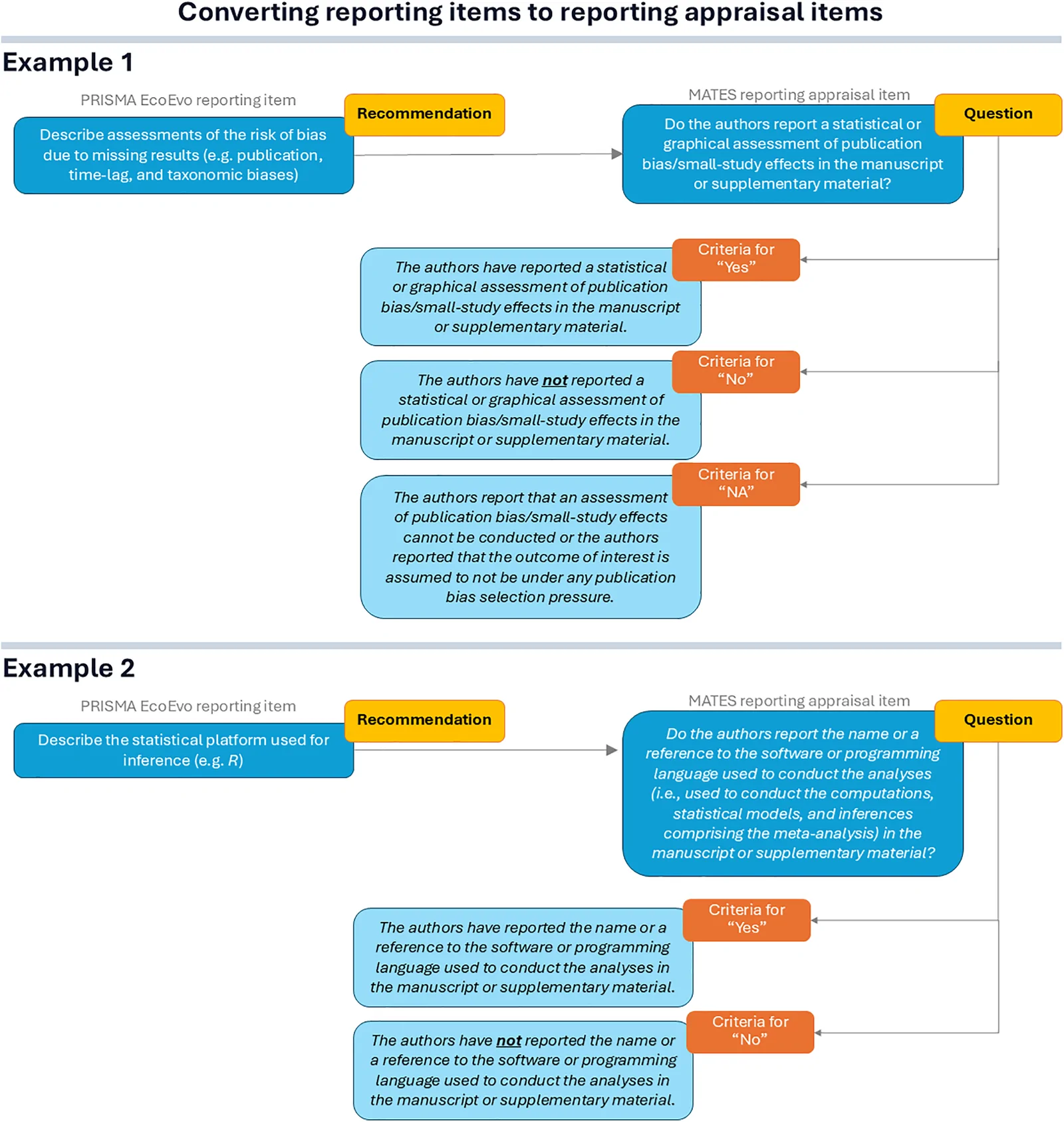
A conceptual diagram illustrating how to convert a reporting item to a reporting appraisal tool with a clear question and assessment criteria. Examples of reporting items have been taken from PRISMA 2020 [4] and PRISMA-EcoEvo [18], and reporting appraisal items have been taken from MATES [7].“NA” is an abbreviation of Not Applicable.

## Rule 7: Evaluate the tool’s repeatability and usability

A step often overlooked in reporting guidelines—but essential for a reporting appraisal tool—is the evaluation of both repeatability (the consistency of ratings) and usability (the practicality of applying the tool) [6]. This step is critical because the appraisal tool should be suitable for repeated use, and its outcomes should be comparable across different appraisers. To assess repeatability and usability, a replication exercise should be conducted on a defined set of studies [10]. As a minimal viable validation exercise, developers can recruit three to five independent appraisers to apply the tool to a small, purposively selected sample of 5–10 manuscripts, recording both time-to-completion per manuscript and inter-rater agreement; this modest exercise is sufficient to flag ambiguous items and unworkable time burdens before wider dissemination. Metrics commonly used to assess repeatability include percentage agreement and chance-corrected statistics such as Cohen’s Kappa [16] and Gwet’s AC1 [17]. Cohen’s Kappa can behave paradoxically when item prevalence is very high or very low—that is, when most appraisers agree an item is fulfilled or unfulfilled—producing a low Kappa value despite high observed agreement. Gwet’s AC1 was developed to address this limitation and provides more stable estimates under such conditions. We therefore recommend Gwet’s AC1 as a useful complement to Cohen’s Kappa for reporting appraisal tools, where most items are typically fulfilled or unfulfilled by the majority of manuscripts, while Cohen’s Kappa remains appropriate for items with more balanced (near 50/50) prevalence of fulfilment. In addition to between-appraiser agreement, within-appraiser consistency can also be evaluated by having the same reviewer re-rate the same studies after an appropriate time interval (test–retest). Although usability is harder to measure comprehensively, practical indicators include the time required to complete the appraisal and user feedback on item clarity and interpretability. Together, repeatability and usability metrics help prospective users determine whether the tool is suitable for their specific context and constraints.“

## Rule 8: Report the decision-making process

Maintaining trust in a reporting appraisal tool requires transparent, well-documented decision-making throughout its development [3]. To support this transparency, developers should provide a clear summary of the feedback received at each stage of tool development and describe how this input informed subsequent revisions to the reporting items [2]. Although systematically capturing and synthesising expert and community input is resource‑intensive, even concise stage‑by‑stage summaries demonstrate transparency and responsiveness in the tool’s development process. The feedback provided throughout the Delphi-like process can then be shared alongside the final appraisal tool in the manuscript either in the supplementary materials or in a dedicated document [19]. This level of clear documentation will build trust in the appraisal tool and support future updates, adaptations, and re-validation.

## Rule 9: Provide clear guidance for use of the tool

After documenting the development process, provide clear guidance on how the appraisal tool is intended to be used [8]. Such guidance may include recommended use cases, detailed explanations of each item, and illustrative examples of good reporting practices [20]. This elaborative material can be presented alongside the tool itself or as a separate Explanation and Elaboration Document [20]. These instructions should be informed by feedback from those who completed the validation exercise, as they will have firsthand insight into which elements require additional clarification beyond the reporting item itself. Providing explicit and clear instructions helps users understand the intent of each item and in turn enhances the tool’s repeatability and usability.

## Rule 10: Plan effective presentation and dissemination

The final step involves preparing a statement paper and a dissemination plan [8]. This includes determining the format in which the tool will be presented. For example, this may involve deciding whether to provide the checklist in a text file, a spreadsheet, or both [4,5]. Next, the core team should decide how to present the elaboration and methodological details, e.g., in the supplementary material or as separate companion papers [4]. Dissemination may include co‑publication across journals, stakeholder endorsements, and translation to enhance accessibility and uptake [8]. Finally, the dissemination plan should include mechanisms for gathering continued feedback and implementing periodic updates to ensure the appraisal tool remains current and relevant.

## Conclusions

In this work, we have sought to distil the key principles for developing a reporting appraisal tool, covering the full pathway from early conceptualisation to tool preparation and dissemination planning. However, understanding these principles is only the starting point; developing a robust reporting appraisal tool is inherently complex, iterative, and resource-intensive. Because such tools are intended for repeated use and shape judgments about reporting completeness, their development should be grounded in consensus-based methods that integrate the perspectives of both experts and the broader research community. The purpose of reporting appraisal tools is not to place an additional burden on authors, but to provide meta-scientists, editors, and reviewers with a repeatable and usable tool for assessing reporting completeness. Ultimately, the strongest tools are built the long way: through an open, transparent, fair, and democratic process.

### Declaration of generative AI and AI-assisted technologies

While preparing this manuscript, the authors used OpenAI’s GPT-5.4, to enhance the clarity, readability, and flow of drafts originally written by the authors. The authors take full responsibility for the content included in this work.

## Supporting information

S1 Table An overview of the 10 simple rules for developing a reporting appraisal tool. (PDF)
