# Supplementary material for: Ten simple rules for developing a reporting appraisal tool
Source: PLoS Comput Biol. 2026 Oct 16;22(10):e1014841. doi: 10.1371/journal.pcbi.1014841 (PMC13674756; doi:10.1371/journal.pcbi.1014841)
Supplement: Supplementary file 1 [file pcbi.1014841.s001.pdf]

Ten simple rules for developing a reporting appraisal tool

Item	Description	Example
Rule 1: Define reporting completeness as a distinct type of methodological quality	Reporting appraisal tools assess whether a manuscript sufficiently describes what was done, distinct from tools that judge methodological quality or risk of bias.	A reporting appraisal tool checks whether eligibility criteria are reported, not whether they were well designed.
Rule 2: Assemble a steering group to guide development	Form a core team with methodological expertise, topic knowledge, and practical insight to define scope, design the development process, and oversee feedback and revisions.	The steering group coordinates the process, documents key decisions, and later leads preparation of the manuscript and supporting materials.
Rule 3: Select a suitable reporting guideline as a foundation	Specify the field, study type, and design of interest, then identify a closely matching reporting guideline to supply candidate items, using resources like the EQUATOR Network or existing item banks.	If no guideline closely matches, developers may adapt or extend an existing one to add items specific to their needs.
Rule 4: Incorporate diverse expert and community opinion	Engage methodological experts, stakeholders, and community members across relevant PROGRESS-Plus factors to ensure feedback is well-rounded and representative.	Including participants from low- and middle-income countries can surface whether a candidate item is feasible to mandate in resource-limited settings.
Rule 5: Gather feedback iteratively using complementary methods	Use a Delphi-like process combining surveys and consensus meetings, with pre-specified item-retention thresholds (e.g., 70% agreement) and a prioritisation round to filter candidate items.	A steering group sets a 70% consensus threshold before the Delphi process begins, then uses forced ranking or a total-item cap to trim the pool.
Rule 6: Design the structure of the reporting appraisal tool	Convert guideline statements into direct, answerable questions with explicit fulfilment criteria, using scaled scoring for graded requirements and binary scoring for discrete elements.	“Report eligibility criteria” becomes the scaled question “Does the manuscript state inclusion and exclusion criteria?” (yes/partial/no), or the binary “Does the manuscript state the eligibility criteria?” (yes/no).

Item	Description	Example
Rule 7: Evaluate the tool's repeatability and usability	Assess repeatability (consistency of ratings) and usability (practicality of use) via a small validation exercise with three to five appraisers rating five to ten manuscripts, recording inter-rater agreement and time-to-completion.	Gwet's AC1 is recommended alongside Cohen's Kappa, since AC1 gives more stable estimates when most appraisers agree an item is fulfilled or unfulfilled.
Rule 8: Report the decision-making process	Provide a clear, stage-by-stage summary of feedback received during development and how it shaped revisions to the reporting items.	Feedback from the Delphi-like process is shared alongside the final tool in supplementary materials or a dedicated document to build trust and support future updates.
Rule 9: Provide clear guidance for use of the tool	Offer guidance on intended use cases, item explanations, and examples of good reporting practice, informed by feedback from the validation exercise.	This material may appear alongside the tool itself or as a separate Explanation and Elaboration Document clarifying items appraisers found unclear.
Rule 10: Plan effective presentation and dissemination	Decide on the tool's format (e.g., text file, spreadsheet), how methodological detail will be presented (supplementary material vs. companion papers), and a dissemination plan with mechanisms for periodic updates.	Dissemination may include co-publication across journals, stakeholder endorsements, and translation to support accessibility and uptake.
